# Trends in Avoidable Hospitalizations Before and During the COVID-19 Pandemic: Multiple Cross-Sectional Study Using Administrative Data From Beijing, China

**DOI:** 10.2196/69768

**Published:** 2025-07-03

**Authors:** Xiangzhen Wang, Yin Chen, Yuqi Ta, Moning Guo, Hongqiao Fu

**Affiliations:** 1 School of Public Health, Peking University, No. 38 Xueyuan Road, Beijing, 100191, China, 86 15120079272; 2 Beijing Municipal Health Big Data and Policy Research Center, Beijing, China; 3 School of Public Finance and Taxation, Central University of Finance and Economics, Beijing, China

**Keywords:** avoidable hospitalization, COVID-19, primary care, accessibility, quality of care

## Abstract

**Background:**

Avoidable hospitalizations (AHs) have been widely used in high-income countries as a proxy indicator for the quality of primary care. However, it is rarely evaluated in low- and middle-income countries such as China. Studies examining changes in AHs before and during the COVID-19 pandemic are also limited. The appropriateness of AHs as an indicator measuring primary care quality under pandemic conditions has not been well discussed.

**Objective:**

This study aims to describe trends in AHs in Beijing, China, during both the prepandemic (2016-2019) and pandemic (2020-2021) periods and examine factors associated with AH rates.

**Methods:**

We used hospital discharge data of Beijing residents between January 1, 2016, and December 31, 2021. We identified AH cases from all discharge cases and calculated AH rates each year, adjusting for population structure changes. We performed regression analyses to explore factors associated with AH rates, where the COVID-19 outbreak, health care resources, and socioeconomic characteristics were used as the main explanatory variables.

**Results:**

Before the COVID-19 pandemic, the total number of hospital discharges in Beijing increased steadily from 2016 to 2019 but decreased sharply in 2020 and partially rebounded in 2021. The sex- and age-standardized AH rate per 100,000 population rose from 514.7 (95% CI 511.4‐517.9) in 2016 to 552.8 (95% CI 549.4‐556.1) in 2019. Then it declined to 331.2 (95% CI 328.6‐333.8) in 2020 and rebounded to 465.1 (95% CI 462.1‐468.1) in 2021, which was still below the prepandemic level. Regression analyses show that the presence of newly confirmed COVID-19 cases was significantly associated with a lower AH rate. As for other factors, higher densities of primary physicians were linked to lower AH rates. Moreover, AH rates were also associated with population structure, the level of economic development, and demographic variables.

**Conclusions:**

The AH rate in Beijing exhibited a consistent upward trend before the pandemic and remained higher than in many high-income countries. These characteristics suggest a potential overuse of tertiary care and highlight the necessity for health care system reforms in Beijing, particularly a transition from the hospital-centered model to a primary care–focused delivery system. In addition, the observed associations between AH rates and factors, such as pandemic shock and socioeconomic variables, indicate that AH should be interpreted with appropriate controls when it is used as an indicator of primary care performance.

## Introduction

In the past 2 decades, improving access to high-quality primary or ambulatory care has been a global policy priority [1]. It has been regarded as a means to improve population health and control health expenditures. One key measure that policy makers and researchers use to evaluate the accessibility to high-quality primary care is hospitalizations related to ambulatory care sensitive conditions (ACSCs), as they could be potentially avoided with timely and effective health care management [2,3]. These ACSCs-related hospitalizations are often referred to as avoidable hospitalizations (AHs) that may impose substantial burdens on individuals, the health care system, and the whole society.

AHs have been widely used to evaluate primary care performance in high-income countries, such as the United States, Europe, Australia, and South Korea [4-9]. A series of studies have also identified factors associated with AHs in high-income countries, such as access to primary care, organizational arrangements, and individual socioeconomic status [10-12]. However, to the best of our knowledge, few studies have investigated the trends in AHs and their associated factors in low- and middle-income countries [13,14], where health care resources are generally insufficient and the quality of care is usually unsatisfactory. More evidence on AHs from low-income countries contributes to a better understanding of their health care system performance through international comparisons.

Furthermore, our understanding of trends in AHs during the COVID-19 pandemic is limited, especially in low-income countries. A series of studies on high-income countries have shown the impacts of the COVID-19 pandemic on disrupting access to non–COVID-19–related medical services, such as decreasing hospitalizations and preventive service usage [15-17]. However, little is known about changes in AHs in low- and middle-income countries before and during the pandemic, although existing studies have found substantial declines in outpatient visits and hospital admissions [18-20]. A few studies have also examined changes in admissions for ACSCs during the pandemic but results have been inconsistent [21-24]. On the one hand, lockdown policies during the pandemic may reduce hospitalizations related to ACSCs, resulting in a decrease in the AH rate. On the other hand, delayed health care usage may exacerbate ACSCs, leading to an increased AH rate.

In this study, we aim to use Beijing as an example to examine AHs in the setting of the Chinese health care system. We used hospital discharge data from Beijing between 2016 and 2021 to describe trends in AHs, which cover periods prior to and during the COVID-19 outbreak. We also performed regression analysis to explore the factors influencing AH rates, with COVID-19 cases and socioeconomic characteristics as the main explanatory variables. As the capital of China, Beijing leads in socioeconomic development and population health in China. It is the medical hub of North China with abundant medical resources. The social health insurance schemes in Beijing, especially in the coverage of outpatient care, are more generous than those in most other Chinese cities. The hospital discharge rate among Beijing’s local residents in 2019 was approximately 11.1% (2,431,274/21,901,000), which was lower than both the national average in China (18.6%) and the average across 35 Organisation for Economic Cooperation and Development (OECD) countries (14.9%) [25,26]. However, similar to other Chinese cities, Beijing has a profit-driven, hospital-centered health care system with weak primary care [27-29]. In 2019, more than 50.0% of the medical revenues of public hospitals in Beijing came from pharmaceuticals and diagnostic tests, while less than 25.0% were from medical services [30]. Approximately 172.1 million outpatient visits were provided by hospitals, while primary care institutions accounted for only 68.3 million visits [31]. Moreover, 62.6% of total health expenditures went to hospitals, while only 10.2% were allocated to primary health care institutions [32]. All these figures suggested the system’s heavy reliance on hospital-based care. Due to low perceived quality, patients do not trust primary care facilities much and often bypass the primary care system. Consequently, they often seek care at tertiary hospitals for simple conditions, leading to overcrowded tertiary hospitals and rising health expenditures. Therefore, results about AHs in Beijing not only inform us of the health system performance in Beijing but also provide implications for the challenges in the Chinese health care system.

One of the prominent events during our study period was the outbreak of COVID-19 pandemic, which may profoundly affect the trend and level of AHs. After the outbreak of the COVID-19 pandemic in January 2020, the Beijing municipal government took strict measures to control the viral transmission, including restrictions on human mobility, body temperature monitoring, and contact tracing [33]. Although there were only sporadic outbreaks and a small number of confirmed cases in the following 2 years (as shown in Figures S1 and S2 in Multimedia Appendix 1), these strict measures may generate substantial effects on people’s daily lives, including consumption behaviors, willingness to work from home, investment decisions, and health care usage [34-37]. As some studies have already examined the complex drivers of AHs (eg, primary care quality, socioeconomic conditions, hospital admission policy, and health insurance types) and questioned the appropriateness of AHs in measuring the quality of primary care [38,39], trends in AHs before and during the COVID-19 pandemic could provide important implications for the effectiveness and limitations of the usage of AH as a proxy for access and quality of primary care, given that large shocks to both the demand and supply sides of health care services were caused by the COVID-19 pandemic.

## Methods

### Study Population and Data Source

In this study, we mainly used hospital discharge data in Beijing between January 1, 2016, and December 31, 2021, to investigate trends in AHs. This dataset was acquired from the Beijing Municipal Health Big Data and Policy Research Center. The dataset included all hospital discharge records in 267 hospitals in Beijing, except for 13 military hospitals. It contained extensive information on medical diagnoses (the *ICD-10* [*International Statistical Classification of Diseases, Tenth Revision*] code), procedures (the *ICD-9-CM-3* [Volume 3 of the *International Classification of Diseases, Ninth Revision, Clinical Modification*] code), length of stay, health expenditures, health insurance types, and patient demographic characteristics [40]. As this dataset encompassed both the prepandemic and pandemic periods, it allowed us to scrutinize how the COVID-19 pandemic affected health care usage among residents in Beijing.

**Figure 1.**
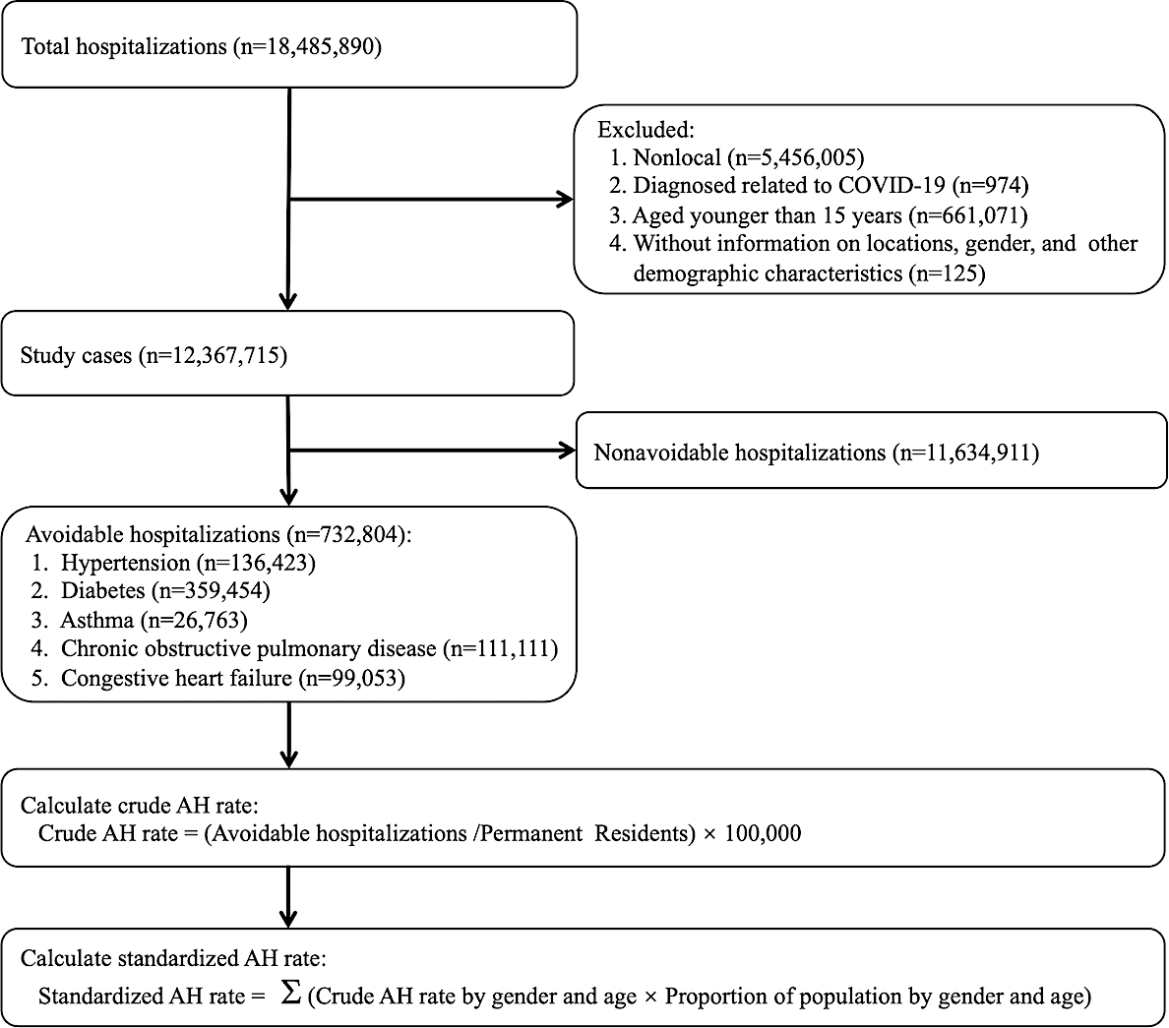
Flowchart for identifying AHs and calculating AH rates. The initial dataset contained 18,485,890 discharge cases during the period between 2016 and 2021. Then, we narrowed our sample to local patients because this study aimed to examine AHs among permanent residents in Beijing. Beijing is the largest medical hub in China, serving a large share of inpatient cases from various provinces nationwide. Since these nonlocal inpatients’ conditions tend to be more severe and are not related to primary care in Beijing, nonlocal inpatients were excluded to avoid biasing the estimation of AH rates. We also excluded inpatient cases related to COVID-19, cases related to age less than 15 years [41], and cases lacking information on residential locations, sex, and other demographic characteristics. Finally, we obtained a sample of 12,367,715 cases for our analysis. AH: Avoidable hospitalization.

According to the Health Care Quality Indicator Definitions proposed by the OECD [41], discharges with a principle of *ICD-10-CM* (*International Classification of Diseases, Tenth Revision, Clinical Modification*) code for any 1 of the following 6 ACSCs were identified as AHs: hypertension, diabetes, asthma, chronic obstructive pulmonary disease (COPD), congestive heart failure (CHF), and diabetes-related lower limb amputation. Because of their small volume (less than 200 cases per year) and the overlap with diabetes cases, diabetes-related lower limb amputations were merged into the scope of diabetes for analysis. This classification allowed us to compare our results with international studies as well as some other China-specific findings [13,42,43]. As depicted in Figure 1, we identified a total of 732,804 cases of AHs, adhering to the specific inclusion and exclusion criteria outlined in Table S1 in Multimedia Appendix 1. Among these AHs, there were 136,423 cases of hypertension, 359,454 cases of diabetes, 26,763 cases of asthma, 111,111 cases of COPD, and 99,053 cases of CHF.

### Variables

In this study, we defined key, dependent variables at both case and district levels. At the case level, the dependent variable is a binary indicator denoting whether a given hospital admission was an AH. At the district level, the dependent variable is the monthly AH rate for each district. As shown in Figure 1, we conducted calculations by dividing the count of AHs by the total resident population and then multiplied the result by 100,000 to derive the crude AH rates per 100,000 population. In addition, we calculated sex- and age-standardized AH rates, referencing the population structure of Beijing in 2010, to examine the consistency and stability of our findings.

Regarding independent variables, we also collected data at both the individual and district levels. First, we extracted demographic and clinical characteristics from hospital discharge records, including sex, age, marital status, health insurance types, and the Charlson Comorbidity Index (CCI) score. Second, we collected data on socioeconomic and health resources at the district level to examine the associated factors of AH rates. These variables included densities of hospital physicians (ie, the number of hospital physicians per 1000 population), primary physicians (ie, the number of primary physicians per 1000 population), proportion of males, proportion of the population aged older than 65 years, education level, and economic development, which were calculated based on information from the Beijing annual reports of medical institutions and Beijing statistical yearbooks. Finally, we obtained data on the daily confirmed COVID-19 cases in Beijing from the Chinese Center for Disease Control and Prevention to construct 2 dummy variables to indicate the presence of the COVID-19 pandemic in the current month or the previous month within each district of Beijing. Table S2 in Multimedia Appendix 1 shows the definitions of all variables used in this study.

### Statistical Analysis

We first conducted descriptive analyses. Categorical variables are described with numbers and percentages, and continuous variables are described with means and SDs. We reported crude and standardized AH rates with 95% CIs for years between 2016 and 2021 and used chi-square tests to examine the statistical significance of differences between different years. In addition, we calculated AH rates across various districts in Beijing to show geographic variations. There are 16 administrative districts in Beijing, consisting of 6 urban districts located at the center of the city and 10 suburban districts. Compared with the suburban districts, the central districts have better socioeconomic developments, higher population density, and more abundant medical resources.

Furthermore, we performed linear regressions with robust SEs to explore the associated factors of AHs. First, we used district-level AH data to explore the association between AHs and the COVID-19 outbreak, health care resources, and socioeconomic characteristics. The model can be written as follows:


(1) $$Y_{it}=\beta_{0}+\beta_{1}{COVID}_{it}+\beta_{2}Resource_{it}+\beta_{3}Controls_{it}+\lambda_{year}+\epsilon_{it}$$

where the subscript *i* denotes the district in Beijing and *t* denotes each month from January 2016 to December 2021. The dependent variable *Y_it_* is the crude or standardized monthly AH rate for each district. The independent variable COVID*_it_* is a dummy variable that takes 1 if the district experienced 1 or more newly confirmed COVID-19 cases in the current month. Otherwise, it equals 0. Resource*_it_* denotes variables measuring the density of medical resources at the district level, including the number of hospital physicians per 1000 population and the number of primary physicians per 1000 population. Because of collinearity, other variables measuring the density of medical resources, such as hospital beds per 1000 population, are not included in the analysis. Controls*_it_* are a series of socioeconomic variables, including population structure, average education level, and economic developments. *λ*_year_ denotes the year fixed effects to control unobservable variables that change over time but are constant over districts. *ϵ_it_* denotes the robust error term.

In addition, we used individual-level data to perform logistic regressions to explore individual-level factors associated with AHs. The model can be written as follows:


(2) $$Pr\left(Y_{ijst}=1\right)=\frac{1}{1+e^{-\left(\beta_{0}+\beta_{1}{COVID}_{ijst}+\beta_{2}X_{ijst}+\beta_{3}{Hospital}_{jt}+\beta_{4}{District}_{st}+\lambda_{t}\right)}}$$

where the subscript *i* denotes the hospitalized case, *j* denotes the hospital, *s* denotes the district in Beijing, and *t* indicates the year. The dependent variable *Y_ijst_* is a binary indicator that takes 1 if the hospitalized patient case *i* who resides in district *s* was admitted to hospital *j* in year *t* for ACSCs, and 0 if the admission was due to other conditions. The independent variable COVID*_ijst_* is a dummy variable that takes 1 if the district where the patient lived had 1 or more new confirmed COVID-19 cases within a period before the patient was admitted. Otherwise, it equals 0. *X_ijst_* denotes a series of individual-level variables, including sex, age, marital status, CCI score, and health insurance types. Hospital*_jt_* denotes the ranking of the hospital, which is classified into 3 groups: primary, secondary, or tertiary hospitals. District*_st_* denotes variables measuring the density of medical resources in the district where the patient lived, including the number of hospital physicians per 1000 population, the number of primary physicians per 1000 population, and other socioeconomic variables. *λ*_t_ denotes the year fixed effects to control for unobservable variables that change over time but are constant over the patient. Robust SEs are clustered at the district level. All statistical analyses were carried out in Stata 17.0 (StataCorp LLC). All *P* values were from 2-sided tests and results were deemed statistically significant at *P*<.1.

### Ethical Considerations

This study was approved as a secondary use of administrative data with a waiver of informed consent by the Peking University institutional review board. All data used in the study were deidentified prior to analysis to protect participants' privacy and confidentiality. The study was conducted in accordance with institutional ethical standards and followed the STROBE (Strengthening the Reporting of Observational Studies in Epidemiology) reporting guideline for cross-sectional studies.

## Results

### Summary Statistics

Table 1 shows the summary statistics for variables in the discharge data. From 2016 to 2019, the number of discharge cases for local inpatients aged older than 15 years increased from 1,931,230 to 2,303,308, while it decreased to 1,697,228 in 2020 and rebounded to 2,282,250 in 2021. The proportion of females was consistently higher than that of males and it decreased modestly over the years. The share of inpatients older than 60 years increased from 46.0% (887,617/1,931,230) in 2016 to 50.5% (1,151,718/2,282,250) in 2021. The share of inpatients without comorbidities as measured by the CCI (Table S3 in Multimedia Appendix 1) decreased by about 6 percentage points during this period. Patients receiving treatment at tertiary hospitals took a larger share after 2020, which implies a significant change in the patient composition during the COVID-19 pandemic. Additionally, these findings were in line with rising expenditures per admission after 2020, compared with those between 2016 and 2019.

Tables S4 and S5 in Multimedia Appendix 1 show the summary statistics for AHs and non-AHs, respectively. Similar to the trends in all inpatient cases, both the numbers of AHs and non-AHs experienced large declines during the pandemic. However, the reduction in AHs (38.4%, 54,499/141,811) in 2020 was markedly greater than that in non-AHs (25.5%, 555,581/2,161,497). More than half of AHs were males, contrary to the sex share among non-AHs. The proportion of AHs aged older than 60 years was consistently larger than that of non-AHs during our study period. Most AHs had comorbidities and the group of AHs had much higher CCI scores than non-AHs. However, the average expenditures for AHs were consistently lower than those for non-AHs.

**Table 1.** Summary statistics of all study cases^a^.

Variables	2016	2017	2018	2019	2020	2021
Total study cases	1,931,230	2,010,201	2,143,498	2,303,308	1,697,228	2,282,250
AHs^b^, n (%)	118,764 (6.1)	125,712 (6.3)	134,928 (6.3)	141,811 (6.2)	87,312 (5.1)	124,277 (5.4)
Non-AHs^b^, n (%)	1,812,466 (93.9)	1,884,489 (93.7)	2,008,570 (93.7)	2,161,497 (93.8)	1,609,916 (94.9)	2,157,973 (94.6)
Sex, n (%)						
Male	804,176 (41.6)	857,130 (42.6)	928,838 (43.3)	1,001,061 (43.5)	743,233 (43.8)	1,022,609 (44.8)
Female	1,127,054 (58.4)	1,153,071 (57.4)	1,214,660 (56.7)	1,302,247 (56.5)	953,995 (56.2)	1,259,641 (55.2)
Age (years), n (%)						
15‐39	552,136 (28.6)	535,717 (26.7)	532,211 (24.8)	556,681 (24.2)	417,628 (24.6)	506,023 (22.2)
40‐59	491,477 (25.5)	515,863 (25.7)	550,768 (25.7)	584,500 (25.4)	437,957 (25.8)	624,509 (27.4)
60‐79	647,796 (33.5)	697,979 (34.7)	775,375 (36.2)	856,302 (37.2)	639,299 (37.7)	899,392 (39.4)
≥80	239,821 (12.4)	260,642 (13.0)	285,144 (13.3)	305,825 (13.3)	202,344 (11.9)	252,326 (11.1)
Marital status, n (%)						
Unmarried^c^	194,987 (10.1)	197,506 (9.8)	224,798 (10.5)	259,816 (11.3)	203,233 (12.0)	296,146 (13.0)
Married	1,736,243 (89.9)	1,812,695 (90.2)	1,918,700 (89.5)	2,043,492 (88.7)	1,493,995 (88.0)	1,986,104 (87.0)
CCI score^d^, n (%)						
0	1,019,696 (52.8)	1,028,803 (51.2)	1,054,523 (49.2)	1,110,311 (48.2)	810,073 (47.7)	1,067,047 (46.8)
1‐2	494,200 (25.6)	524,864 (26.1)	574,434 (26.8)	620,420 (26.9)	446,545 (26.3)	609,370 (26.7)
3‐4	262,134 (13.6)	282,375 (14.1)	314,127 (14.7)	343,646 (14.9)	245,645 (14.5)	337,759 (14.8)
≥5	155,200 (8.0)	174,159 (8.7)	200,414 (9.4)	228,931 (9.9)	194,965 (11.5)	268,074 (11.8)
Health insurance types, n (%)						
UEBMI^e^	1,130,824 (58.6)	1,214,368 (60.4)	1,339,584 (62.5)	1,484,728 (64.5)	1,112,266 (65.5)	1,547,479 (67.8)
URBMI^f^ or NCRMS^g^	314,991 (16.3)	326,698 (16.3)	314,739 (14.7)	319,374 (13.9)	228,482 (13.5)	312,375 (13.7)
Others^h^	485,415 (25.1)	469,135 (23.3)	489,175 (22.8)	499,206 (21.7)	356,480 (21.0)	422,396 (18.5)
Hospital ranking, n (%)						
Primary	9297 (0.5)	13,231 (0.7)	10,220 (0.5)	7547 (0.3)	3988 (0.2)	3906 (0.2)
Secondary	516,936 (26.8)	503,479 (25.1)	521,391 (24.3)	515,976 (22.4)	375,445 (22.1)	308,888 (13.5)
Tertiary	1,404,997 (72.8)	1,493,491 (74.3)	1,611,887 (75.2)	1,779,785 (77.3)	1,317,795 (77.6)	1,969,456 (86.3)
Expenditure, mean (SD), ¥^i^	17,469 (27,212)	18,192 (28,105)	18,756 (29,171)	18,944 (28,808)	22,060 (34,955)	21,015 (33,301)
LoS^j^ (day), mean (SD)	10.8 (47.8)	11.2 (84.7)	10.5 (41.6)	9.6 (30.9)	10.1 (36.6)	9.2 (44.0)

a Categorical variables are described with numbers and percentages (%), and continuous variables are described with means and SDs.

b AHs: avoidable hospitalizations.

c Unmarried status includes never married, widowed, and divorced.

d CCI score: the Charlson Comorbidity Index score. It was included to measure the severity of comorbidities, which was calculated for each case based on the information from the hospital discharge data (Table S3 in Multimedia Appendix 1). It was further categorized into 4 subgroups (0, 1‐2, 3‐4, and ≥5) [44].

e UEBMI: urban employee basic medical insurance.

f URBMI: urban resident basic medical insurance.

g NCRMS: new rural cooperative medical system. URBMI and NCRMS, mainly provided for minors and unemployed persons, were put together for analysis because they had been merged since 2018.

h Other types of health insurance schemes include commercial medical insurance, other social insurance, poverty relief, public free health, and without insurance coverage.

i All expenditures are reported in Chinese yuan (CNY). The average exchange rate in 2021 was 6.4515 CNY to 1 USD.

j LoS: length of stay.

### Trends in AHs

The first row of Table 2 shows crude changes in overall AHs in Beijing between 2016 and 2021, suggesting that the COVID-19 pandemic had significant impacts on AHs. Before the pandemic (2016-2019), the overall crude AH rate per 100,000 population exhibited a consistent upward trend, rising annually from the lowest of 633.4 (95% CI 629.8‐637.0) in 2016 to the highest of 740.0 (95% CI 736.2‐743.8) in 2019. The difference in the crude AH rate between 2016 and 2019 was statistically significant (difference=106.6; *P*<.001). However, during the pandemic, the crude AH rate experienced a sharp decline to 452.4 (95% CI 449.4‐455.4) in 2020 and rebounded to 639.3 (95% CI 635.8‐642.9) in 2021, but the crude AH rate in 2021 was still lower than the average level in the prepandemic period. Sex- and age-standardized AH rates followed a similar trend to crude AH rates before and during the pandemic. As for regional differences, Figure S3 and Table S6 in Multimedia Appendix 1 report AH rates at the district level from 2016 to 2021. Overall, suburban districts consistently have higher AH rates than central districts, showing similar trends. Figure S4 in Multimedia Appendix 1 also illustrates trends in mean expenditure and length of stay for avoidable hospitalizations from 2016 to 2021.

**Table 2.** Trends of avoidable hospitalization rates in Beijing, 2016‐2021.

Variable	AH^a^ rates per 100,000 population (95% CI)						2016 versus 2019		2019 versus 2021		2016 versus 2021	
	2016	2017	2018	2019	2020	2021	Difference^b^	*P* value	Difference	*P* value	Difference	*P* value
Crude rates												
Total AHs	633.4 (629.8‐637.0)	665.5 (661.9‐669.2)	709.2 (705.4‐712.9)	740 (736.2‐743.8)	452.4 (449.4‐455.4)	639.3 (635.8‐642.9)	106.6	<.001	–100.7	<.001	5.9	.02
Hypertension	119 (117.4‐120.5)	126.5 (124.8‐128.1)	132.9 (131.3‐134.6)	138.6 (137.0‐140.3)	77.9 (76.6‐79.1)	120.1 (118.5‐121.6)	19.7	<.001	–18.6	<.001	1.1	.32
Diabetes	301.8 (299.3‐304.3)	319.5 (316.9‐322.0)	348 (345.4‐350.7)	365.4 (362.7‐368.1)	222.3 (220.2‐224.4)	326 (323.5‐328.5)	63.5	<.001	–39.4	<.001	24.2	<.001
Asthma	28.2 (27.4‐29.0)	26.5 (25.7‐27.2)	26.1 (25.4‐26.8)	25 (24.3‐25.7)	14.9 (14.4‐15.4)	19.7 (19.1‐20.4)	–3.2	<.001	–5.3	<.001	–8.5	<.001
COPD^c^	111.1 (109.6‐112.6)	112.3 (110.8‐113.8)	108.7 (107.2‐110.2)	111.8 (110.3‐113.3)	63 (61.9‐64.1)	76.2 (75.0‐77.4)	0.7	.50	–35.6	<.001	–34.9	<.001
CHF^d^	73.3 (72.1‐74.5)	80.9 (79.6‐82.2)	93.4 (92.0‐94.8)	99.1 (97.7‐100.6)	74.3 (73.1‐75.5)	97.3 (95.9‐98.7)	25.9	<.001	–1.8	.07	24.0	<.001
Standardized rates^e^												
Total AHs	514.7 (511.4‐517.9)	524.7 (521.5‐528.0)	542.2 (538.9‐545.5)	552.8 (549.4‐556.1)	331.2 (328.6‐333.8)	465.1 (462.1‐468.1)	38.1	<.001	–87.7	<.001	–49.6	<.001
Hypertension	97.9 (96.5‐99.3)	101.2 (99.8‐102.6)	103.7 (102.2‐105.1)	106 (104.6‐107.5)	58.3 (57.2‐59.4)	90.2 (88.9‐91.6)	8.1	<.001	–15.8	<.001	–7.7	<.001
Diabetes	254.3 (252.0‐256.6)	262.2 (259.9‐264.5)	277.8 (275.4‐280.1)	286.3 (283.9‐288.7)	172.2 (170.3‐174.0)	249.6 (247.4‐251.9)	32.0	<.001	–36.7	<.001	–4.7	.004
Asthma	23.3 (22.6‐24.0)	21.3 (20.7‐22.0)	20.5 (19.8‐21.1)	19.3 (18.7‐20.0)	11.4 (10.9‐11.8)	14.9 (14.4‐15.4)	–4.0	<.001	–4.4	<.001	–8.4	<.001
COPD	84.2 (82.9‐85.6)	82.2 (80.9‐83.5)	76.5 (75.3‐77.8)	75.8 (74.5‐77.0)	41.3 (40.4‐42.2)	49.4 (48.4‐50.3)	–8.5	<.001	–26.4	<.001	–34.9	<.001
CHF	54.9 (53.8‐55.9)	57.9 (56.8‐58.9)	63.8 (62.6‐64.9)	65.3 (64.2‐66.5)	48 (47.0‐49.0)	61 (59.9‐62.1)	10.5	<.001	–4.4	<.001	6.1	<.001

a AH: avoidable hospitalization.

b Chi-square tests were used to test the statistical significance of differences between different survey years.

c COPD: chronic obstructive pulmonary disease.

d CHF: congestive heart failure.

e Standardized AH rates were standardized by the sex and age population structure of Beijing in 2010.

### Impact of the COVID-19 Pandemic and Associated Factors of AHs

Table 3 reports linear regression results that investigated the impact of the COVID-19 pandemic on the monthly AH rates for each district as well as associated factors of AH rates at the district level. In these regression analyses, we included the key variables COVID_this month_ and COVID_last month_, representing the existence of newly confirmed COVID-19 cases in the district for the current month and the previous month, respectively. We also included variables related to medical resources, population structure, and other control variables. In column 1 of Table 3, results show that the existence of the COVID-19 pandemic in the current month was significantly associated with a decrease in the crude AH rate for that month. Considering that the existence of the COVID-19 pandemic in the previous month might influence the change in AHs in the current month, we further included COVID_last month_ to examine the impact of the pandemic on AHs. In column 2, it is evident that the existence of the COVID-19 pandemic was correlated with a significant decrease in the AH rate in the following month. The magnitude of change caused by the pandemic in the last month was larger than that in the current month. Regarding district-level medical resources, a higher density of hospital physicians was positively associated with AH rates. In contrast, a higher density of primary physicians was associated with lower AH rates. To account for demographic changes in Beijing, we changed the dependent variable from the crude AH rate to the standardized AH rate by the Beijing reference population. As shown in columns 3 and 4 of Table 3, the coefficients for various variables remained largely unchanged when using the standardized AH rate as the dependent variable. When we controlled for month-year fixed effects, our results remained largely robust, as shown in Table S8 in Multimedia Appendix 1.

We performed logistic regression analyses using individual-level data to further test the robustness of our findings. As shown in the first 2 columns of Table S7 in Multimedia Appendix 1, the presence of newly confirmed COVID-19 cases within 14 days or 1 month before hospital admission, as well as a greater number of primary care physicians in the patient’s residential district, was associated with a decreased likelihood of the hospitalization being classified as an AH. These results are consistent with the findings based on district-level data. In terms of individual-level factors, hospitalized patients who were male, older, and had comorbid conditions had a higher probability of being classified as AH cases than their female, younger, and noncomorbid counterparts. Inpatients covered by urban resident basic medical insurance or new rural cooperative medical scheme were more likely to be AHs than those covered by urban employee basic medical insurance. Additionally, patients admitted to primary hospitals were more likely to be AHs than those treated in secondary or tertiary hospitals. Table S7 in Multimedia Appendix 1 also shows regression results using expenditure and length of stay as dependent variables to examine potential changes in case composition.

**Table 3.** The impact of the COVID-19 pandemic on avoidable hospitalization.

Variables	Crude AH^a^ rate^b^				Standardized AH rate^c^			
	Model 1		Model 2		Model 3		Model 4	
	β (RSE)^d^	*P* value	β (RSE)	*P* value	β (RSE)	*P* value	β (RSE)	*P* value
COVID_this month_^e^ (reference no.)								
Yes	−7.752 (3.1325)	.01	−4.234 (2.6500)	.11	−6.928 (2.1914)	.002	−4.151 (1.8445)	.03
COVID_last month_^e^ (reference no.)								
Yes	—^f^	—	−18.873 (2.7642)	<.001	—	—	−14.452 (1.9310)	<.001
Medical resource								
Hospital physicians/1000	2.792 (0.5864)	<.001	2.718 (0.5747)	<.001	3.723 (0.4351)	<.001	3.589 (0.4311)	<.001
Primary physicians/1000	−14.900 (3.3889)	<.001	−13.769 (3.3667)	<.001	−8.638 (2.7418)	.002	−8.153 (2.7280)	.003
Population structure								
Proportion of males	−8.081 (1.0309)	<.001	−8.530 (1.0156)	<.001	—	—	—	—
									Proportion of those aged ≥65 years	1.788 (0.6637)	.007	1.401 (0.6516)	.03	—	—	—	—
Mean educational years	−3.082 (1.7056)	.07	−2.940 (1.6636)	.08	−0.890 (1.1810)	.45	−0.461 (1.1639)	.69
GDP_district_	−0.004 (0.0005)	<.001	−0.004 (0.0005)	<.001	−0.002 (0.0004)	<.001	−0.002 (0.0004)	<.001
Constant	505.081 (75.5535)	<.001	531.931 (74.0819)	<.001	59.399 (15.3988)	<.001	54.670 (15.1962)	<.001
Year fixed effect	Yes	—	Yes	—	Yes	—	Yes	—
Observations	1152	—	1152	—	1152	—	1152	—

a AH: avoidable hospitalization.

b Crude AH rate in models (1) and (2) indicates the monthly crude AH rate of each district.

c Standardized AH rate in models (3) and (4) indicates the standardized monthly AH rate of each district according to the population structure of Beijing in 2010.

d RSE: robust standard error.

e Key variables COVID_this month_ and COVID_last month_ indicate whether new COVID-19 cases were recognized in the district this or last month.

f Not included in this model.

## Discussion

### Principal Findings

Using discharge data from Beijing, this study found that both the crude and the sex- and age-standardized AH rates in Beijing displayed an upward trend before the COVID-19 pandemic (2016‐2019) and experienced a sharp decline after the outbreak of the pandemic. This trend suggests substantial impacts of the COVID-19 pandemic on health care usage among residents in Beijing. Moreover, regression results showed that AHs were significantly associated with multiple factors, including pandemic shocks, densities of health care resources, socioeconomic variables, and various demographic characteristics.

We found that both the crude and the sex- and age-standardized AH rates in Beijing displayed an upward trend before the COVID-19 pandemic (2016‐2019) and experienced a sharp decline after the outbreak of the pandemic. This trend suggests substantial impacts of the COVID-19 pandemic on health care usage among residents in Beijing.

Based on the results regarding trends in AHs before and during the COVID-19 pandemic, we further compared the AH rate in Beijing with that in some high-income countries, including the United States, the United Kingdom, France, Canada, and Korea, by calculating sex- and age-standardized AH rates based on the OECD 2015 reference population [45]. As shown in Table 4, the overall sex- and age-standardized AH rate per 100,000 population in Beijing was 904.1 in 2019, higher than that in the United Kingdom (530.5 in 2019), France (636.0 in 2015), Canada (537.3 in 2019), and Korea (641.0 in 2019), but slightly lower than that in the United States (961.0 in 2019). This comparison suggests that it is necessary for Beijing to strengthen its primary care system and restructure its hospital-centered delivery system.

In terms of specific diseases, the sex- and age-standardized AH rates for hypertension and diabetes in Beijing were much higher than those in all comparison countries. That finding may be due to the relatively low awareness and control rates of these conditions among the Chinese population [29], which often results in delayed diagnosis and more severe symptoms requiring hospitalization. In addition, medical insurance policies in China provide higher reimbursement rates for inpatient care than for outpatient care, which may incentivize hospitalization over outpatient care. By contrast, the standardized AH rates for asthma, COPD, and CHF in Beijing were at a relatively low level, compared with those in other countries. Given the high proportion of hypertension and diabetes among all AHs in Beijing, more attention should be paid to the prevention and control of hypertension and diabetes.

Moreover, this study showed that relatively high-income districts with abundant primary care medical resources generally had lower AH rates than suburban districts. This was consistent with the regression result that a higher density of primary physicians was associated with a lower AH rate. It also implied that the AH rate could at least partially reflect the accessibility to primary care. In addition, we found that the AH rate was significantly associated with demographic and socioeconomic variables, such as population structure and economic development. These supported the previous finding that AHs should be interpreted in conjunction with metrics of demographic composition and the level of economic development [39,46].

More importantly, we found that the COVID-19 pandemic was associated with the reduction of the AH rates in Beijing. This association remained robust across alternative regression specifications, including using various definitions of the COVID-19 pandemic, changing the dependent variable from crude rates to standardized rates, and using data at the individual level. These results highlight that we cannot interpret solely the reduction in AH rates as the result of the changing performance of primary health care, as the pandemic has caused many changes in health care providers’ and patients’ behaviors that could be related to the AH rates. During the COVID-19 pandemic, many countries took large-scale public health measures such as stay-at-home orders, closing schools, cancellation of mass gatherings, and even lockdowns of endemic areas [20,47-50]. In Beijing, despite low COVID-19 mortality and a limited number of confirmed cases, the government implemented a stringent “zero-COVID” policy, including movement restrictions, community lockdowns, testing mandates, and hospital visitor bans. These measures created substantial barriers to hospital access for patients with no COVID-19 cases. Compared to non-AHs, AH patients typically present with milder symptoms and some of them seek hospitalization primarily because the reimbursement rate for inpatient care is higher than for outpatient care in the nonpandemic period. As a result, during the pandemic period, AH patients became more sensitive to the demand-side factors (eg, patients with ACSC avoiding hospitals unless their symptoms became severe) and the supply-side factors (eg, providers restricting admissions for mild cases to prevent in-hospital viral transmission), which collectively contributed to the larger reductions in AHs.

Taken together, our findings highlight the complexity of using AH as a proxy for access to primary care, especially when there are systemwide changes. The determinants of AHs are multifactorial, including factors at the patient level, regional level, and national system level. These factors may not be independent but intertwined, especially when there are changes that simultaneously occur in both the demand and supply for medical care. As a result, when we observe a change in AH rates, it may be inappropriate and inaccurate to interpret them solely as the consequence of changes in the accessibility and quality of primary health care or outpatient care.

**Table 4.** International comparisons of sex- and age-standardized avoidable hospitalization rate^a^.

Variables	Beijing (2019)	United States^b^ (2019)	United Kingdom^b^ (2019)	France^b^ (2015)	Canada^b^ (2019)	Korea^b^ (2019)
Total AHs^c^	904.1	961.0	530.5	636.0	537.3	641.0
Hypertension	167.5	60.6	18.7	34.8	14.6	79.2
Diabetes	405.1	248.5	81.7	155.6	98.9	237.3
Asthma	29.4	37.3	74.9	29.8	13.9	68.5
COPD^d^	153.5	186.8	239.2	128.8	229.5	161.5
CHF^e^	148.5	427.8	116.0	287.0	180.4	94.5

a All the sex- and age-standardized AH rates per 100,000 population were standardized by the OECD (Organisation for Economic Co-operation and Development) 2015 reference population.

b These countries’ sex- and age-standardized AH rates were obtained from the OECD website.

c AHs: avoidable hospitalizations.

d COPD: chronic obstructive pulmonary disease.

e CHF: congestive heart failure.

### Limitations

There are several limitations to this study. First, we can only roughly measure the impact of primary health care access but not directly measure the effect of primary health care quality on AHs due to data availability. Second, we cannot evaluate the gains in social welfare from reductions in AHs during the pandemic. We also cannot clearly identify the mechanisms through which the pandemic significantly reduced the AH rate. Third, we used the definition of ACSCs proposed by OECD, which might not fully capture China’s epidemiological features and health system characteristics [51]. Finally, due to the limited time span of the data, we were unable to explore long-term changes in AH rates after the COVID-19 pandemic, which may help us better understand the long-lasting effects of pandemic-related disruptions in health care usage. These limitations should be addressed in future research.

### Conclusions

Using administrative discharge data, we found that Beijing had a relatively high AH rate compared with other high-income countries. Trend analysis showed that the overall AH rate in Beijing displayed an upward trend between 2016 and 2019 but experienced a sharp decrease in 2020. The increasing AH rates in Beijing before the pandemic highlight the necessity to strengthen its primary care system and restructure its hospital-centered delivery system in Beijing. Potential strategies include improving the capacity of primary care physicians, creating incentives for health care providers to provide preventive and primary care, and expanding the benefit coverage of health insurance schemes for outpatient services. Regression results showed that AHs were significantly associated with multiple factors, including the COVID-19 pandemic shocks, the regional density of health care resources, and demographic variables. These results imply that changes in AHs cannot solely be interpreted as changes in access and quality of primary care. Appropriate controls accounting for the multifactorial drivers of all hospital admissions are needed when we use this indicator as a tool to monitor health care system performance, especially when there are system-wide changes such as the COVID-19 pandemic.

## Supplementary material

10.2196/69768 Multimedia Appendix 1 Additional statistical analyses, data sources, and inclusion and exclusion criteria for study cases.

## Abbreviations

ACSC: ambulatory care sensitive condition
AH: avoidable hospitalization
CCI: Charlson Comorbidity Index
CHF: congestive heart failure
COPD: chronic obstructive pulmonary disease
*ICD-10*: *International Statistical Classification of Diseases, Tenth Revision*
*ICD-10-CM*: *International Classification of Diseases, Tenth Revision, Clinical Modification*
*ICD-9-CM-3*: Volume 3 of the *International Classification of Diseases, Ninth Revision, Clinical Modification*
OECD: Organisation for Economic Cooperation and Development
STROBE: Strengthening the Reporting of Observational Studies in Epidemiology
