# Supplementary material for: Trends in Avoidable Hospitalizations Before and During the COVID-19 Pandemic: Multiple Cross-Sectional Study Using Administrative Data From Beijing, China
Source: JMIR Public Health Surveill. 2025 Jul 3;11:e69768. doi: 10.2196/69768 (PMC12244741; doi:10.2196/69768)
Supplement: Multimedia Appendix 1 [file publichealth-v11-e69768-s001.docx]

# *Figures*

**Figure S1.** Weekly new confirmed COVID-19 cases in Beijing, 2020-2021. The x-axis of this graph denotes the weeks between 2020 and 2021 (for example: 2020w1 means the 1^st^ week in 2020 and 2020w27 means the 27^th^ week in 2020). The y-axis denotes the number of newly confirmed COVID-19 cases for one week. Data source: Chinese Center for Disease Control and Prevention (China CDC).


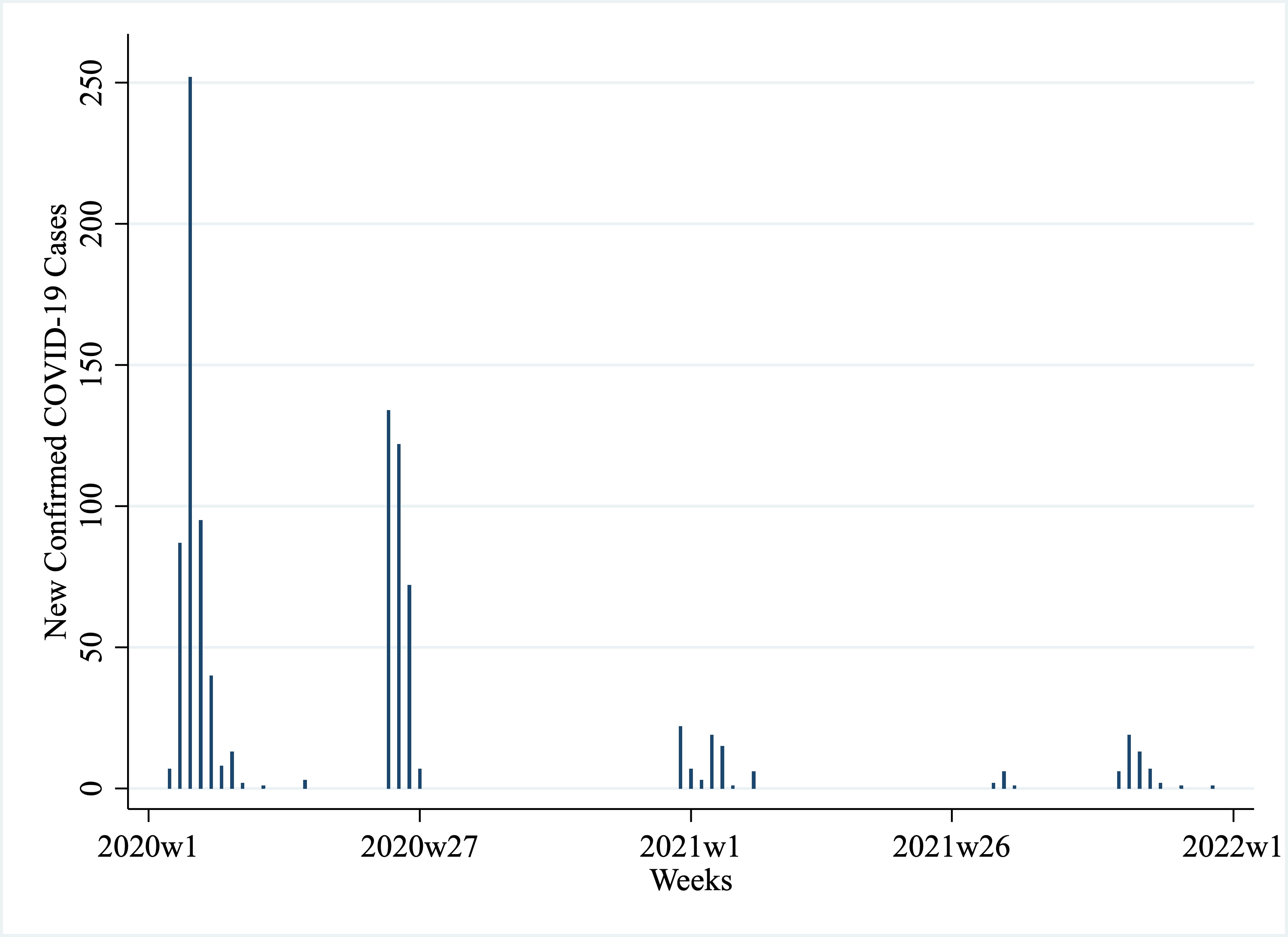


**Figure S2.** Regional disparities of new confirmed COVID-19 cases in Beijing, 2020-2021. Subfigures a, b, c, and d display each district’s new confirmed COVID-19 cases during periods from January to June 2020, from July to December 2020, from January to June 2021, and from July to December 2021, respectively. Data source: Chinese Center for Disease Control and Prevention (China CDC).

**Figure S3.** Regional disparities of the sex- and age-standardized AH rates in Beijing, 2016-2021. Rates in this figure were all standardized according to the population structure of Beijing in 2010. AH: avoidable hospitalization.


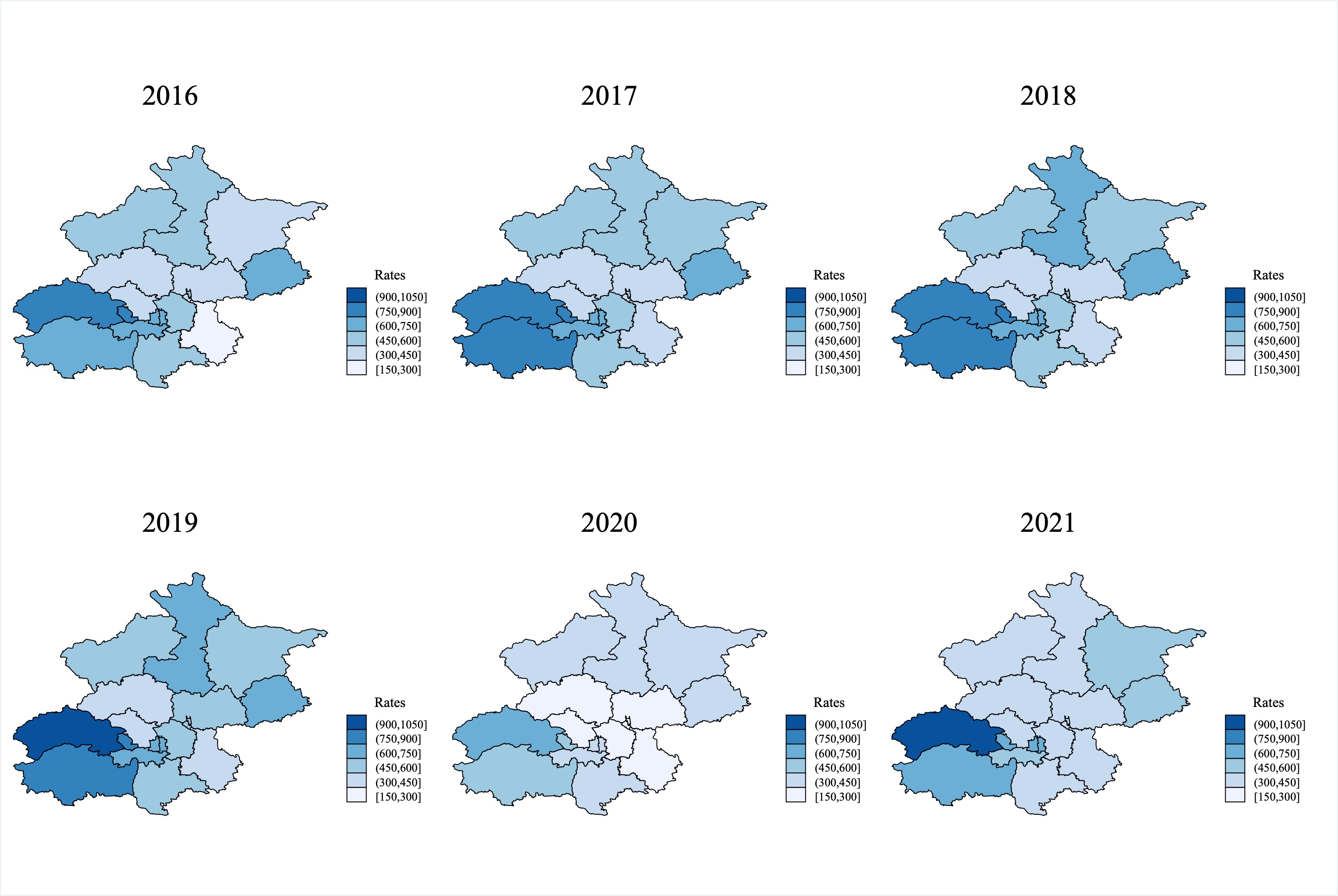


**Figure S4.** Trends of expenditure and length of stay on avoidable hospitalizations. Subfigure a shows the trends of expenditure per admission from 2016 to 2021 and subfigure b shows the trends of hospital length of stay per admission from 2016 to 2021. AH: avoidable hospitalization; COPD: chronic obstructive pulmonary disease; CHF: congestive heart failure.

***Tables***

**Table S1.** Inclusion and exclusion criteria.

| **Conditions** | **Coverage** | **Inclusion criteria** | **Exclusion criteria** |
| --- | --- | --- | --- |
| Hypertension | Local hospitalization aged 15 and older | 1. Principal diagnosis codes: I10, I11.9, I12.9, I13.9 2. Non-maternal/non-neonatal hospital admissions | 1. Cases where the patient died in hospital during the admission. 2. Cases resulting from a transfer from another acute care institution. 3. Cases with cardiac procedure codes in any field. 4. Cases with specified pregnancy, childbirth, and puerperium codes in any field. 5. Cases that are same day/day only admissions. |
| Diabetes | Local hospitalization aged 15 and older | 1. Principal diagnosis codes: E10.0-E10.9, E11.0-E11.9, E13.0-E13.9, E14.0-E14.9 2. Non-maternal/non-neonatal hospital admissions | 1. Cases where the patient died in hospital during the admission. 2. Cases resulting from a transfer from another acute care institution. 3. Cases with specified pregnancy, childbirth, and puerperium codes in any field. 4. Cases that are same day/day only admissions. |
| Asthma | Local hospitalization aged 15 and older | 1. Principal diagnosis codes: J45.0, J45.1, J45.8, J45.9, J46 2. Non-maternal/non-neonatal hospital admissions | 1. Cases where the patient died in hospital during the admission. 2. Cases resulting from a transfer from another acute care institution. 3. Cases with specified pregnancy, childbirth, and puerperium codes in any field. 4. Cases with cystic fibrosis and anomalies of the respiratory system diagnosis code in any field. 5. Cases that are same day/day only admissions. |

Notes: This inclusion and exclusion criteria referred to Health Care Quality Indicator Definitions proposed by the Organization for Economic Cooperation and Development.

**Table S1.** Inclusion and exclusion criteria (continued)

| **Conditions** | **Coverage** | **Inclusion criteria** | **Exclusion criteria** |
| --- | --- | --- | --- |
| Chronic obstructive pulmonary disease (COPD) | Local hospitalization aged 15 and older | 1. Principal diagnosis codes: J40, J41.1, J41.8, J42, J43.0, J43.1, J43.2, J43.8, J44.0, J44.8, J44.9, J47 2. Non-maternal/non-neonatal hospital admissions | 1. Cases where the patient died in hospital during the admission. 2. Cases resulting from a transfer from another acute care institution. 3. Cases with specified pregnancy, childbirth, and puerperium codes in any field. 4. Cases that are same day/day only admissions. |
| Congestive heart failure (CHF) | Local hospitalization aged 15 and older | 1. Principal diagnosis codes: I11.0, I13.0, I13.2, I50.0, I50.1, I50.9   2. Non-maternal/non-neonatal hospital admissions | 1. Cases where the patient died in hospital during the admission.  2. Cases resulting from a transfer from another acute care institution.  3. Cases with cardiac procedure codes in any field.  4. Cases with specified pregnancy, childbirth, and puerperium codes in any field.  5. Cases that are same day/day only admissions. |

Notes: This inclusion and exclusion criteria referred to Health Care Quality Indicator Definitions proposed by the Organization for Economic Cooperation and Development

**Table S2.** Data source and definitions of variables.

| **Data** | **Data source** | **Variables** | **Definition** |
| --- | --- | --- | --- |
| Hospital Discharge Data | Beijing Municipal Health Big Data and Policy Research Center | Sex | Male=0, Female=1 |
|  |  | Age | ≥15 years |
|  |  | Marital status | Unmarried, Married |
|  |  | CCI score groups | 0, 1-2, 3-4, ≥5 |
|  |  | Principal diagnosis codes | According to ICD-10 |
|  |  | Secondary diagnosis codes | According to ICD-10 |
|  |  | Surgical and operational codes | According to ICD-9-CM-3 |
|  |  | Health insurance types | Urban employee basic medical insurance (UEBMI),  Urban resident basic medical insurance (NRBMI),  New rural cooperative medical scheme (NRCMS), and Others |
| Annual Reports of Medical Institution | Beijing Municipal Health Big Data and Policy Research Center | Hospital ranking | Primary, secondary, and tertiary |
|  |  | Hospital physicians | The number of physicians in hospital |
|  |  | Primary physicians | The number of physicians in primary medical institutions |
| Statistic Yearbook | Beijing Municipal Bureau of Statistics | Residents | The number of permanent residents |
| COVID-19 Data | Center for Disease Control and Prevention | COVID_this_month_, COVID_last_month_ | Whether there had new confirmed COVID-19 cases in the district the current month or the previous month |
| Beijing Regional Statistical Yearbook | Beijing Municipal Bureau of Statistics | GDP | Gross regional domestic product, Chinese yuan |
|  |  | Aging population share | Proportion of the population aged over 65 years old |
| 2020 China population census | National Bureau of Statistics | Education level | Years of education per resident |

**Table S3.** Charlson Comorbidities Index (CCI) score.

| **CCI item** | **Score** | **ICD-10 codes** |
| --- | --- | --- |
| Myocardial infarction | 1 | I21, I22, I252 |
| Congestive heart failure | 1 | I110, I130, I132, I50 |
| Peripheral vascular disease | 1 | I72-I73, I77, K55 |
| Cerebrovascular disease | 1 | G45, G46, I60-I69 |
| Dementia | 1 | F01-F06, G30, G31, R54 |
| Chronic pulmonary disease | 1 | J40-J44, J47 |
| Connective tissue disease | 1 | M05, M06, M315, M32-M34, M351, M353, M360 |
| Ulcer disease | 1 | K25-K28 |
| Mild liver disease | 1 | B15-B19, K71, K3, K75 |
| Diabetes mellitus | 1 | E10.9, E11.9, E12.9, E13.9, E14.9 |
| Hemiplegia | 2 | G80-G83 |
| Moderate or severe renal disease | 2 | N18.3-N18.6, Z94 |
| Diabetes mellitus with end organ damage | 2 | E10.1-E10.8, E11.1-E11.8, E12.1-E12.8, E13.1-E13.8, E14.1-E14.8 |
| Malignant tumor | 2 | C81-C85 |
| Leukemia | 2 | C90-C96 |
| Lymphoma | 2 | D46 |
| Moderate or severe liver disease | 3 | K72-K77 |
| Metastatic solid tumor | 6 | C77-C79 |
| Acquired immunodeficiency syndrome | 6 | B20-B24 |

**Table S4.** Summary statistics of AHs in Beijing, 2016-2021.

| Variables | 2016 | 2017 | 2018 | 2019 | 2020 | 2021 |
| --- | --- | --- | --- | --- | --- | --- |
| **Total AHs** | 118,764 | 125,712 | 134,928 | 141,811 | 87,312 | 124,277 |
| **Gender** |  |  |  |  |  |  |
| Male | 62,987 | 67,181 | 72,131 | 75,986 | 47,251 | 66,818 |
|  | (53.0%) | (53.4%) | (53.5%) | (53.6%) | (54.1%) | (53.8%) |
| Female | 55,777 | 58,531 | 62,797 | 65,825 | 40,061 | 57,459 |
|  | (47.0%) | (46.6%) | (46.5%) | (46.4%) | (45.9%) | (46.2%) |
| **Age** |  |  |  |  |  |  |
| 15–39 | 7,422 | 8,468 | 9,067 | 9,861 | 6,633 | 9,501 |
|  | (6.3%) | (6.7%) | (6.7%) | (7.0%) | (7.6%) | (7.7%) |
| 40–59 | 34,538 | 35,503 | 37,780 | 38,824 | 23,051 | 34,454 |
|  | (29.1%) | (28.2%) | (28.0%) | (27.4%) | (26.4%) | (27.7%) |
| 60–79 | 52,902 | 55,973 | 60,358 | 64,146 | 39,570 | 57,794 |
|  | (44.5%) | (44.5%) | (44.7%) | (45.2%) | (45.3%) | (46.5%) |
| ≥80 | 23,902 | 25,768 | 27,723 | 28,980 | 18,058 | 22,528 |
|  | (20.1%) | (20.5%) | (20.6%) | (20.4%) | (20.7%) | (18.1%) |
| **Marital status** |  |  |  |  |  |  |
| Unmarried^c^ | 10,702 | 10,565 | 12,968 | 15,262 | 10,278 | 15,276 |
|  | (9.0%) | (8.4%) | (9.6%) | (10.8%) | (11.8%) | (12.3%) |
| Married | 108,062 | 115,147 | 121,960 | 126,549 | 77,034 | 109,001 |
|  | (91.0%) | (91.6%) | (90.4%) | (89.2%) | (88.2%) | (87.7%) |
| **CCI score**^d^ |  |  |  |  |  |  |
| 0 | 14,887 | 15,503 | 15,180 | 15,010 | 9,942 | 14,128 |
|  | (12.5%) | (12.3%) | (11.3%) | (10.6%) | (11.4%) | (11.4%) |
| 1–2 | 45,856 | 48,705 | 52,067 | 54,875 | 34,924 | 47,982 |
|  | (38.6%) | (38.7%) | (38.6%) | (38.7%) | (40.0%) | (38.6%) |
| 3–4 | 39,310 | 41,798 | 46,286 | 49,469 | 29,625 | 43,043 |
|  | (33.1%) | (33.3%) | (34.3%) | (34.9%) | (33.9%) | (34.6%) |
| ≥5 | 18,711 | 19,706 | 21,395 | 22,457 | 12,821 | 19,124 |
|  | (15.8%) | (15.7%) | (15.9%) | (15.8%) | (14.7%) | (15.4%) |
| **Health insurance types** |  |  |  |  |  |  |
| UEBMI^e^ | 76,368 | 81,532 | 88,487 | 93,783 | 57,912 | 86,978 |
|  | (64.3%) | (64.9%) | (65.6%) | (66.1%) | (66.3%) | (70.0%) |
| URBMI^f^ or NCRMS^g^ | 25,179 | 25,686 | 24,909 | 26,324 | 15,770 | 21,596 |
|  | (21.2%) | (20.4%) | (18.5%) | (18.6%) | (18.1%) | (17.4%) |
| Others^h^ | 17,217 | 18,494 | 21,532 | 21,704 | 13,630 | 15,703 |
|  | (14.5%) | (14.7%) | (16.0%) | (15.3%) | (15.6%) | (12.6%) |
| **Hospital ranking** |  |  |  |  |  |  |
| Primary | 910 | 1,402 | 1,003 | 265 | 110 | 139 |
|  | (0.8%) | (1.1%) | (0.7%) | (0.2%) | (0.1%) | (0.1%) |
| Secondary | 32,931 | 32,231 | 34,288 | 34,966 | 22,098 | 18,950 |
|  | (27.7%) | (25.6%) | (25.4%) | (24.7%) | (25.3%) | (15.3%) |
| Tertiary | 84,923 | 92,079 | 99,637 | 106,580 | 65,104 | 105,188 |
|  | (71.5%) | (73.3%) | (73.8%) | (75.2%) | (74.6%) | (84.6%) |
| **Expenditure, mean (SD**^i^**), ¥** | 13,753.3 | 13,993.8 | 14,039.0 | 13,870.7 | 15,582.7 | 14,681.5 |
|  | (14390.3) | (14646.5) | (13398.4) | (13811.3) | (19484.6) | (17435.0) |
| **LoS**^j^**, mean (SD), day** | 12.1 | 11.9 | 11.7 | 11.2 | 11.6 | 10.6 |
|  | (14.8) | (17.9) | (23.1) | (14.3) | (16.6) | (10.2) |

^a^ In this table, categorical variables are described with numbers and percentages (%), and continuous variables are described with means and standard deviations (SDs).

^b^ AHs: avoidable hospitalizations.

^c^ Unmarried status includes never married, widowed, and divorced.

^d^ CCI score: the Charlson Comorbidity Index score. It was included to measure the severity of comorbidities, which was calculated for each case based on the information from the hospital discharge data (see Appendix Table S3). It was further categorized into four subgroups (0, 1–2, 3–4, and ≥5) [44].

^e^ UEBMI: urban employee basic medical insurance.

^f^ URBMI: urban resident basic medical insurance.

^g^ NCRMS: new rural cooperative medical system. URBMI and NCRMS, mainly provided for minors and unemployed persons, were put together for analysis because they had been merged since 2018.

^h^ Other types of health insurance schemes include commercial medical insurance, other social insurance, poverty relief, public free health, and without insurance coverage.

^i^ SD: standard deviations.

^j^ LoS: length of stay.

**Table S5.** Summary statistics of non-AHs in Beijing, 2016-2021.

| Variables | 2016 | 2017 | 2018 | 2019 | 2020 | 2021 |
| --- | --- | --- | --- | --- | --- | --- |
| **Total non-AHs** | 1,812,466 | 1,884,489 | 2,008,570 | 2,161,497 | 1,609,916 | 2,157,973 |
| **Gender** |  |  |  |  |  |  |
| Male | 741,189 | 789,949 | 856,707 | 925,075 | 695,982 | 955,791 |
|  | (40.9%) | (41.9%) | (42.7%) | (42.8%) | (43.2%) | (44.3%) |
| Female | 1,071,277 | 1,094,540 | 1,151,863 | 1,236,422 | 913,934 | 1,202,182 |
|  | (59.1%) | (58.1%) | (57.4%) | (57.2%) | (56.8%) | (55.7%) |
| **Age** |  |  |  |  |  |  |
| 15–39 | 544,714 | 527,249 | 523,144 | 546,820 | 410,995 | 496,522 |
|  | (30.1%) | (28.0%) | (26.1%) | (25.3%) | (25.5%) | (23.0%) |
| 40–59 | 456,939 | 480,360 | 512,988 | 545,676 | 414,906 | 590,055 |
|  | (25.2%) | (25.5%) | (25.5%) | (25.3%) | (25.8%) | (27.3%) |
| 60–79 | 594,894 | 642,006 | 715,017 | 792,156 | 599,729 | 841,598 |
|  | (32.8%) | (34.1%) | (35.6%) | (36.7%) | (37.3%) | (39.0%) |
| ≥80 | 215,919 | 234,874 | 257,421 | 276,845 | 184,286 | 229,798 |
|  | (11.9%) | (12.5%) | (12.8%) | (12.8%) | (11.5%) | (10.7%) |
| **Marital status** |  |  |  |  |  |  |
| Unmarriedc | 184,285 | 186,941 | 211,830 | 244,554 | 192,955 | 280,870 |
|  | (10.2%) | (9.9%) | (10.6%) | (11.3%) | (12.0%) | (13.0%) |
| Married | 1,628,181 | 1,697,548 | 1,796,740 | 1,916,943 | 1,416,961 | 1,877,103 |
|  | (89.8%) | (90.1%) | (89.5%) | (88.7%) | (88.0%) | (87.0%) |
| **CCI scored** |  |  |  |  |  |  |
| 0 | 1,004,809 | 1,013,300 | 1,039,343 | 1,095,301 | 800,131 | 1,052,919 |
|  | (55.4%) | (53.8%) | (51.8%) | (50.7%) | (49.7%) | (48.8%) |
| 1–2 | 448,344 | 476,159 | 522,367 | 565,545 | 411,621 | 561,388 |
|  | (24.7%) | (25.3%) | (26.0%) | (26.2%) | (25.6%) | (26.0%) |
| 3–4 | 222,824 | 240,577 | 267,841 | 294,177 | 216,020 | 294,716 |
|  | (12.3%) | (12.8%) | (13.3%) | (13.6%) | (13.4%) | (13.7%) |
| ≥5 | 136,489 | 154,453 | 179,019 | 206,474 | 182,144 | 248,950 |
|  | (7.5%) | (8.2%) | (8.9%) | (9.6%) | (11.3%) | (11.5%) |
| **Health insurance types** |  |  |  |  |  |  |
| UEBMIe | 1,054,456 | 1,132,836 | 1,251,097 | 1,390,945 | 1,054,354 | 1,460,501 |
|  | (58.2%) | (60.1%) | (62.3%) | (64.4%) | (65.5%) | (67.7%) |
| URBMI^f^ or NCRMS^g^ | 289,812 | 301,012 | 289,830 | 293,050 | 212,712 | 290,779 |
|  | (16.0%) | (16.0%) | (14.4%) | (13.6%) | (13.2%) | (13.5%) |
| Othersh | 468,198 | 450,641 | 467,643 | 477,502 | 342,850 | 406,693 |
|  | (25.8%) | (23.9%) | (23.3%) | (22.1%) | (21.3%) | (18.9%) |
| **Hospital ranking** |  |  |  |  |  |  |
| Primary | 8,387 | 11,829 | 9,217 | 7,282 | 3,878 | 3,767 |
|  | (0.5%) | (0.6%) | (0.5%) | (0.3%) | (0.2%) | (0.2%) |
| Secondary | 484,005 | 471,248 | 487,103 | 481,010 | 353,347 | 289,938 |
|  | (26.7%) | (25.0%) | (24.3%) | (22.3%) | (22.0%) | (13.4%) |
| Tertiary | 1,320,074 | 1,401,412 | 1,512,250 | 1,673,205 | 1,252,691 | 1,864,268 |
|  | (72.8%) | (74.4%) | (75.3%) | (77.4%) | (77.8%) | (86.4%) |
| **Expenditure, mean (SD**^i^**), ¥** | 17,712.6 | 18,472.4 | 19,072.9 | 19,277.3 | 22,411.6 | 21,379.5 |
|  | (27,829.1) | (28,758.3) | (29,907.5) | (29,495.9) | (35,568.6) | (33,954.1) |
| **LoS**^j^**, mean (SD), day** | 10.7 | 11.1 | 10.4 | 9.5 | 10.0 | 9.2 |
|  | (49.2) | (87.4) | (42.5) | (31.7) | (37.4) | (45.2) |

^a^ In this table, categorical variables are described with numbers and percentages (%), and continuous variables are described with means and standard deviations (SDs).

^b^ AHs: avoidable hospitalizations.

^c^ Unmarried status includes never married, widowed, and divorced.

^d^ CCI score: the Charlson Comorbidity Index score. It was included to measure the severity of comorbidities, which was calculated for each case based on the information from the hospital discharge data (see Appendix Table S3). It was further categorized into four subgroups (0, 1–2, 3–4, and ≥5) [44].

^e^ UEBMI: urban employee basic medical insurance.

^f^ URBMI: urban resident basic medical insurance.

^g^ NCRMS: new rural cooperative medical system. URBMI and NCRMS, mainly provided for minors and unemployed persons, were put together for analysis because they had been merged since 2018.

^h^ Other types of health insurance schemes include commercial medical insurance, other social insurance, poverty relief, public free health, and without insurance coverage.

^i^ SD: standard deviations.

^j^ LoS: length of stay.

**Table S6.** AH rates in Beijing by district, 2016-2021.

| Districts | 2016 | 2017 | 2018 | 2019 | 2020 | 2021 |
| --- | --- | --- | --- | --- | --- | --- |
| **Crude rates (per 100,000 population)** | | | | | | |
| Dongcheng | 1107.2 | 1134.1 | 1179.6 | 1250.3 | 775.5 | 1166.2 |
| Xicheng | 996.5 | 1041.7 | 1096.5 | 1159.6 | 666.5 | 1038.3 |
| Chaoyang | 569.0 | 588.2 | 638.7 | 686.8 | 402.8 | 626.1 |
| Fengtai | 842.6 | 915.5 | 990.4 | 1018.0 | 598.8 | 889.7 |
| Shijingshan | 1053.3 | 1106.8 | 1235.9 | 1260.1 | 763.5 | 1089.4 |
| Haidian | 477.4 | 512.9 | 548.8 | 594.4 | 379.5 | 553.1 |
| Mentougou | 1080.9 | 1124.1 | 1292.5 | 1395.0 | 883.4 | 1347.7 |
| Fangshan | 913.4 | 959.3 | 1022.6 | 1043.4 | 696.4 | 836.4 |
| Tongzhou | 326.4 | 376.8 | 381.0 | 385.5 | 258.9 | 386.6 |
| Shunyi | 446.7 | 444.7 | 480.1 | 540.2 | 332.2 | 431.7 |
| Changping | 409.1 | 420.0 | 447.5 | 449.1 | 268.3 | 373.5 |
| Daxing | 489.7 | 506.3 | 527.7 | 539.3 | 335.6 | 450.4 |
| Huairou | 637.5 | 745.8 | 814.5 | 885.4 | 432.0 | 567.6 |
| Pinggu | 1052.3 | 1024.6 | 1054.0 | 1058.1 | 593.1 | 690.2 |
| Miyun | 594.1 | 685.4 | 758.0 | 796.8 | 625.7 | 701.6 |
| Yanqing | 679.9 | 738.2 | 759.5 | 750.4 | 479.4 | 601.9 |
| **Standardized rates (per 100,000 population)**^a^ | | | | | | |
| Dongcheng | 712.0 | 697.1 | 711.7 | 734.9 | 440.9 | 672.8 |
| Xicheng | 640.4 | 652.5 | 667.7 | 680.4 | 384.9 | 616.1 |
| Chaoyang | 463.1 | 463.1 | 483.6 | 506.1 | 288.5 | 441.6 |
| Fengtai | 628.9 | 658.3 | 685.1 | 682.3 | 391.7 | 586.1 |
| Shijingshan | 768.3 | 775.9 | 839.0 | 829.3 | 484.1 | 705.3 |
| Haidian | 409.6 | 420.8 | 432.2 | 449.0 | 274.0 | 402.3 |
| Mentougou | 759.3 | 772.1 | 855.2 | 951.4 | 601.0 | 932.1 |
| Fangshan | 733.6 | 751.5 | 781.4 | 782.4 | 513.7 | 617.5 |
| Tongzhou | 290.1 | 330.3 | 328.1 | 329.3 | 216.7 | 323.2 |
| Shunyi | 397.8 | 388.5 | 414.3 | 461.8 | 281.4 | 361.1 |
| Changping | 424.9 | 422.3 | 434.4 | 426.5 | 249.0 | 339.2 |
| Daxing | 476.0 | 478.3 | 484.3 | 487.9 | 300.3 | 394.3 |
| Huairou | 507.8 | 574.1 | 614.0 | 656.4 | 322.9 | 411.4 |
| Pinggu | 741.4 | 710.7 | 722.2 | 703.4 | 385.8 | 450.7 |
| Miyun | 430.6 | 484.0 | 513.2 | 539.3 | 420.2 | 464.7 |
| Yanqing | 499.1 | 525.5 | 543.7 | 515.4 | 330.9 | 406.9 |

^a^ Standardized rates were standardized by the sex and age population structure of Beijing in 2010.

**Table S7.** Associated factors of avoidable hospitalizations.

| Variables | AH ^a^ or non-AH | | | | Ln (Expenditure) | | LoS^b^ | |
| --- | --- | --- | --- | --- | --- | --- | --- | --- |
|  | (1)^c^ | | (2) ^c^ | | (3) ^c^ | | (4) ^c^ | |
|  | OR  (CRSE)^d^ | *P* value | OR  (CRSE) | *P* value | β  (CRSE) | *P* value | β  (CRSE) | *P* value |
| COVID_14d_^e^ (ref. No) |  |  |  |  |  |  |  |  |
| Yes | 0.846 | <0.001 |  |  | 0.112 | <0.001 | 1.022 | <0.001 |
|  | (0.0075) |  |  |  | (0.0062) |  | (0.0905) |  |
| COVID_1m_^f^ (ref. No) |  |  |  |  |  |  |  |  |
| Yes | —^j^ | — | 0.939 | <0.001 | — | — | — | — |
|  |  |  | (0.0059) |  |  |  |  |  |
| Sex (ref. Male) |  |  |  |  |  |  |  |  |
| Female | 0.919 | <0.001 | 0.919 | <0.001 | -0.046 | <0.001 | -0.337 | <0.001 |
|  | (0.0023) |  | (0.0023) |  | (0.0016) |  | (0.0394) |  |
| Age | 1.005 | <0.001 | 1.005 | <0.001 | 0.013 | <0.001 | 0.098 | <0.001 |
|  | (0.0001) |  | (0.0001) |  | (0.0001) |  | (0.0011) |  |
| Marital status (ref. Unmarried) |  |  |  |  |  |  |  |  |
| Married | 0.998 | 0.69 | 0.998 | 0.71 | -0.085 | <0.001 | -1.599 | <0.001 |
|  | (0.0041) |  | (0.0041) |  | (0.0026) |  | (0.1153) |  |
| CCI score (ref. 0) |  |  |  |  |  |  |  |  |
| 1–2 | 5.918 | <0.001 | 5.919 | <0.001 | 0.232 | <0.001 | 2.199 | <0.001 |
|  | (0.0282) |  | (0.0282) |  | (0.0029) |  | (0.0685) |  |
| 3–4 | 10.180 | <0.001 | 10.180 | <0.001 | 0.247 | <0.001 | 2.526 | <0.001 |
|  | (0.0484) |  | (0.0484) |  | (0.0028) |  | (0.0683) |  |
| ≥5 | 6.596 | <0.001 | 6.595 | <0.001 | 0.214 | <0.001 | 2.373 | <0.001 |
|  | (0.0348) |  | (0.0348) |  | (0.0029) |  | (0.0744) |  |
| Health insurance (ref. UEBMI^g^) |  |  |  |  |  |  |  |  |
| URBMI^h^/NCRMS^i^ | 1.042 | <0.001 | 1.042 | <0.001 | -0.099 | <0.001 | -1.206 | <0.001 |
|  | (0.0035) |  | (0.0035) |  | (0.0019) |  | (0.0328) |  |
| Others | 0.783 | <0.001 | 0.783 | <0.001 | -0.114 | <0.001 | 0.665 | <0.001 |
|  | (0.0028) |  | (0.0028) |  | (0.0028) |  | (0.1155) |  |
| Hospital ranking (ref. Primary) |  |  |  |  |  |  |  |  |
| Secondary | 0.368 | <0.001 | 0.368 | <0.001 | -0.279 | <0.001 | -5.919 | <0.001 |
|  | (0.0071) |  | (0.0071) |  | (0.0105) |  | (0.5178) |  |
| Tertiary | 0.333 | <0.001 | 0.333 | <0.001 | -0.173 | <0.001 | -7.417 | <0.001 |
|  | (0.0064) |  | (0.0064) |  | (0.0104) |  | (0.5123) |  |
| Medical resource |  |  |  |  |  |  |  |  |
| Hospital physicians/1000 | 1.018 | <0.001 | 1.018 | <0.001 | 0.027 | <0.001 | 0.224 | <0.001 |
|  | (0.0008) |  | (0.0008) |  | (0.0005) |  | (0.0148) |  |
| Primary physicians/1000 | 0.885 | <0.001 | 0.883 | <0.001 | -0.081 | <0.001 | -0.131 | 0.04 |
|  | (0.0038) |  | (0.0038) |  | (0.0027) |  | (0.0643) |  |
|  |  |  |  |  |  |  |  |  |
| Constant | 0.043 | <0.001 | 0.043 | <0.001 | 8.636 | <0.001 | 11.177 | <0.001 |
|  | (0.0009) |  | (0.0009) |  | (0.0112) |  | (0.5185) |  |
|  |  |  |  |  |  |  |  |  |
| Year fixed effect | Yes |  | Yes |  | Yes |  | Yes |  |
| Observations | 12,367,715 |  | 12,367,715 |  | 732,804 |  | 732,804 |  |

^a^ AH: avoidable hospitalization.

^b^ LoS: length of stay.

^c^ Models (1) and (2) display logit regression results by odds ratio (OR); Models (3) and (4) display OLS regression results.

^d^ Cluster-robust standard error at the district level.

^e^ COVID_14d_ indicates whether there have been newly confirmed COVID-19 cases in the district where the patient lived within 14 days before the case was admitted to the hospital.

^f^ COVID_1m_ indicates whether there have been newly confirmed COVID-19 cases in the district where the patient lived within 1 month before the case was admitted to the hospital.

^g^ UEBMI: urban employee basic medical insurance.

^h^ URBMI: urban resident basic medical insurance.

^i^ NCRMS: new rural cooperative medical system.

^j^ Not included in this model.

**Table S8.** Robustness check: controlling for month-year fixed effect.

| Variables | Crude AH rate^a^ | | | | Standardized AH rate^b^ | | | |
| --- | --- | --- | --- | --- | --- | --- | --- | --- |
|  | (1) | | (2) | | (3) | | (4) | |
|  | β (RSE)^c^ | *P* value | β (RSE) | *P* value | β (RSE) | *P* value | β (RSE) | *P* value |
| COVID_this_month_^d^ (ref. No) |  |  |  |  |  |  |  |  |
| Yes | -2.621 | 0.3 | 0.625 | 0.80 | -3.286 | 0.08 | -0.675 | 0.69 |
|  | (2.6911) |  | (2.3500) |  | (1.8867) |  | (1.6668) |  |
| COVID_last_month_^d^ (ref. No) |  |  |  |  |  |  |  |  |
| Yes | —^e^ | — | -17.896 | <.001 | — | — | -13.909 | <.001 |
|  |  |  | (2.6227) |  |  |  | (1.8573) |  |
| Medical resource |  |  |  |  |  |  |  |  |
| Hospital physicians/1000 | 2.799 | <.001 | 2.729 | <.001 | 3.751 | <.001 | 3.622 | <.001 |
|  | (0.4957) |  | (0.4826) |  | (0.3891) |  | (0.3833) |  |
| Primary physicians/1000 | -15.107 | <.001 | -14.030 | <.001 | -8.675 | 0.001 | -8.207 | 0.001 |
|  | (2.9665) |  | (2.9160) |  | (2.5048) |  | (2.4789) |  |
| Population structure |  |  |  |  |  |  |  |  |
| Proportion of males | -7.941 | <.001 | -8.370 | <.001 | — | — | — | — |
|  | (0.9118) |  | (0.8881) |  |  |  |  |  |
| Proportion of aged ≥ 65 | 1.908 | <.001 | 1.540 | 0.006 | — | — | — | — |
|  | (0.5783) |  | (0.5598) |  |  |  |  |  |
| Mean educational years | -3.084 | 0.04 | -2.950 | 0.04 | -0.985 | 0.35 | -0.570 | 0.58 |
|  | (1.4833) |  | (1.4374) |  | (1.0595) |  | (1.0354) |  |
| GDP_district_ | -0.004 | <.001 | -0.004 | <.001 | -0.002 | <.001 | -0.002 | <.001 |
|  | (0.0004) |  | (0.0004) |  | (0.0004) |  | (0.0004) |  |
| Constant | 496.115 | <.001 | 521.731 | <.001 | 60.326 | <.001 | 55.758 | <.001 |
|  | (67.3796) |  | (65.4742) |  | (13.8700) |  | (13.5868) |  |
|  |  |  |  |  |  |  |  |  |
| Month-Year fixed effect | Yes |  | Yes |  | Yes |  | Yes |  |
| Observations | 1152 |  | 1152 |  | 1152 |  | 1152 |  |

^a^ Crude AH rate in Models (1) and (2) indicates the monthly crude AH rate of each district.

^b^ Standardized AH rate in Models (3) and (4) indicates the sex- and age-standardized monthly AH rate of each district according to the population structure of Beijing in 2010.

^c^ Robust standard error.

^d^ Key variables *COVID_this_month_* and *COVID_last_month_* representing the existence of newly confirmed COVID-19 cases in the district for the current month and the previous month.

^e^ Not included in this model.
